# Reversible Oxygenation of 2,4-Diaminobutanoic Acid-Co(II) Complexes

**DOI:** 10.1155/2016/8296365

**Published:** 2016-08-25

**Authors:** Xiang Cheng, Yan Huang, Hui Li, Fan Yue, Hongmei Wen, Jide Wang

**Affiliations:** Key Laboratory of Oil and Gas Fine Chemicals, Ministry of Education and Xinjiang Uyghur Autonomous Region, College of Chemistry and Chemical Engineering, Xinjiang University, Urumqi 830046, China

## Abstract

This paper introduces the structural characterization and studies on reversible oxygenation behavior of a new oxygen carrier Co(II)-2,4-diaminobutanoic acid (DABA) complex in aqueous solution. The composition of the oxygenated complex was determined by gas volumetric method, molar ratio method, and mass spectrometry, and the formula of the oxygenated complex was determined to be [Co(DABA)_2_O_2_]. In aqueous solution, the complex can continuously uptake and release dioxygen and exhibit excellent reversibility of oxygenation and deoxygenation ability. This complex can maintain 50% of its original oxygenation capacity after 30 cycles in 24 h and retain 5% of the original oxygenation capacity after more than 260 cycles after 72 h. When a ligand analogue was linked to histidine (His), the new complex exhibited as excellent reversible oxygenation property as His-Co(II) complex. Insight into the relationship between structural detail and oxygenation properties will provide valuable suggestion for a new family of oxygen carriers.

## 1. Introduction

Oxygen carriers are a series of natural/synthesized products that can reversibly bind dioxygen [1]. Studies on oxygen carriers present important implications in revealing the mechanism of dioxygen transfer and storage in vivo, for example, in hemoglobin and myohemoglobin [2–5]. Amino acids can form various metal complexes with Fe(II), Cu(I), Co(II), and other metal ions [1, 6]. These types of metal complexes are active centers of biological macromolecules, such as peptidyl glycine *α*-hydroxylating monooxygenase, dopamine*β*-monooxygenase, myoglobin, and hemoglobin [7–9]. Researches on metal complexes of amino acids can be an aid to prove the biological activities and function mechanisms of these complexes in organisms. The design and synthesis of transition metal complexes with good affinity to dioxygen and long reversible oxygenation life are key to develop natural oxygen carriers. The most commonly used central metal ions are Fe(II), Cu(I), Co(II), and Mn(II) [10–16], and typical ligands include polyamine, Schiff base, amino acids, and porphyrins [14, 15, 17–19]. Certain compounds show good reversible oxygen absorbing performance in solid state [20, 21], at low temperature, or in nonaqueous media [22, 23]. However, most known model compounds exhibit poorly reversible oxygenation behavior in aqueous solution. Only histidine-Co(II) and iodo-histidine-Co(II) [24–26] complexes exhibit excellent reversibility of dioxygen uptake in aqueous solution to date. The lack of accumulation on the relationship between structural details and oxygenation properties leads to a knowledge gap in the development of novel, inexpensive oxygen carriers. We previously supposed that the special structure of histidine-Co(II) is the key to the reversibility of dioxygen uptake [14, 24]. To prove this concept, we designed and selected several histidine ligand analogues, such as *β*-(2-Pyridyl)-*α*-alanine [24] and 2, 3-diaminopropionic acid [27], and investigated the oxygenation of the resulting Co(II) complexes. Both complexes showed excellent reversible oxygenation as histidine-Co(II) and supported our assumption. We then selected UV-Vis spectrophotometry to detect the oxygenation properties of potential oxygen carriers. The technique was selected because of its simplicity and usefulness in investigating oxygenation in a solution, as UV-Vis absorption spectrum of the oxygenated cobalt complex is significantly distinct from its deoxygenated species. Therefore, the oxygenation and reversibility of the complexes can be observed precisely by using UV-Vis spectrophotometry through monitoring color variations.

In this paper, the reversible oxygenation of 2,4-diaminobutanoic acid-Co(II) was investigated by UV-Vis spectra and oxygenmetry in aqueous solution at room temperature. The formation of the oxygenated complex was characterized by UV-Vis spectrophotometry, infrared (IR) spectroscopy, and mass spectrometry (MS), and the composition of the oxygenated complex was determined by molar ratio method and gas volumetric method. The reversible cycles of oxygenation and the oxygenation life of the complex were tested in aqueous solution, and all results demonstrated that the complex exhibited excellent reversibility in its oxygenation. This work represents a shift away from histidine and histidine analogues, which have previously been confirmed to undergo reversible oxygenation/deoxygenation events, and provides a link to biological systems that utilize iron and copper for this purpose in vivo.

## 2. Experimental

### 2.1. Materials

*L*-2,4-Diaminobutanoic acid hydrochloride (DABA·2HCl) and Co(OAc)_2_·4H_2_O were obtained from Shanghai Hanhong Chemical Co. and Shanghai Chemical Regents Co., respectively. The reagents were used without further purification. A CO_2_-free NaOH solution (0.1 mol·dm^−3^) was prepared by standardization with potassium acid phthalate. An HCl solution (0.1 mol·dm^−3^) was prepared from concentrated hydrochloric acid and standardized with anhydrous sodium carbonate. High-purity (99.99%) O_2_ and N_2_ were used.

### 2.2. UV-Vis Spectrophotometry

Sample solutions were determined by UV-Vis spectrophotometry. N_2_ and O_2_ were alternately bubbled into the solution during testing. Optical absorption spectra were recorded at 25°C ± 0.1°C on a Shimadzu UV2450 spectrophotometer using a 1 cm cuvette over the spectral range of 250–600 nm.

### 2.3. IR Spectroscopy

The ligand and complex aqueous solutions (*C* = 1.0 mol·dm^−3^) were analyzed by Bruker EQUINOX 55 liquid IR spectroscopy after air exposure for 1 h. The pH value of the complex solution was adjusted to 10 by adding dilute NaOH solution (0.1 mol·dm^−3^).

### 2.4. Mass Spectrometry Method

MS was performed on a Waters Quattro Premier XE mass spectrometer equipped with an electrospray ionization source (Micromass, Manchester, UK). The mass analyzer was operated in positive ionization mode for all analytes, and the optimized parameters were as follows: source temperature, 120°C; desolvation temperature, 450°C; capillary voltage, 3.5 kV; desolvation gas flow, 400 L·h^−1^; cone voltage, 60 V; cone gas flow, 50 L·h^−1^; collision energy, 20 eV; and multiplier, 650 V. The mass spectra collected in full-scan positive ion mode were obtained by scanning over the mass range of* m/z* 50 to* m/z* 900. Nitrogen (99.999% purity) was used as desolvation and nebulization gas, and ultrapure argon (99.999% purity) was used as collision gas. A full-scan mass spectrum was obtained by flow injection analysis of individual 1.0 × 10^−2 ^mol·dm^−3^ solutions in water. Acetonitrile/water (50 : 50, v/v) was used as the mobile phase. The concentration of DABA and the complex employed for MS determination was 2.0 × 10^−2 ^mol·dm^−3^.

### 2.5. Molar Ratio Method

Eight aliquots of DABA solution (*C*_DABA_ = 4.0 × 10^−4 ^mol·dm^−3^) were separately added to 10.0 mL volumetric flasks at 0.5, 1.0, 1.5, 2.0, 2.5, 3.0, 3.5, and 4.0 mL. About 1.0 mL of 2.0 × 10^−4 ^mol·dm^−3^ Co(II) solution was then added to each flask, and a pH 10 borate buffer solution was added to volume. O_2_ was bubbled into the sample solutions for 10 min. The absorbance of the solutions was determined using a UV2450 spectrophotometer with a 1 cm cuvette at 314 nm.

### 2.6. Gas Volumetric Method

A 10.0 mL of Co(II) (1.0 × 10^−1 ^mol·dm^−3^) and 10.0 mL of DABA (2.0 × 10^−1 ^mol·dm^−3^) were mixed together in a conical flask in a glove box. The aqueous solution was immediately placed on a eudiometer, which had been filled with oxygen in advance. The decreasing volume of oxygen with time was measured until volume remained constant. The final volume of oxygen was then recorded.

### 2.7. Oxygenmetry

The concentration of dissolved O_2_ in solution would be consumed during oxygenation, which displays the oxygenation evolution of the complex. Hence, the concentrations of dissolved O_2_ in solution were measured using a dissolved oxygen meter over a pH range of 2 to 10 at 25°C ± 0.1°C. KCl (0.1 mol·dm^−3^) was used to maintain the solution at a constant ionic strength. A 25.0 mL of 1.2 × 10^−3 ^mol·dm^−3^ ligand solution was transferred to a titration pool and maintained at pH < 2 by adding 2.0 mL of 0.10 mol·dm^−3^ HCl solution to prevent oxygenation prior to testing. Afterward, 25.0 mL of 6.0 × 10^−4 ^mol·dm^−3^ Co(II) solution was added to the pool, and the solution was sealed with 20.0 mL of cyclohexane to prevent air penetration. Therefore, the concentration changes of the dissolved O_2_ in solution are reasonably related to the oxygenation of the Co(DABA)_2_ complex. The diluted NaOH solution was used to adjust the pH values of the solution from 2 to 10, and the O_2_ concentrations in solution were detected at each pH. After the pH reached 10, dilute HCl was added to adjust solution pH values from 10 to 2 and to observe the reversibility of releasing dioxygen from the complex.

### 2.8. Construction of the Absorption (*A*)-pH Curve

25.0 mL of 4.0 × 10^−4 ^mol·dm^−3^ solution was initially adjusted to pH 2 with dilute HCl solution and 25.0 mL of 2.0 × 10^−4 ^mol·dm^−3^ Co(II) solution was added and mixed. The absorption (*A*) of amino acid-cobalt solution was determined by UV-Vis spectrophotometry at the maximum absorption peak (*λ*_max_) of 314 nm. Solution pH was then adjusted from 2 to 12 with dilute NaOH. Absorption *A* was recorded with the variation of pH, and the suitable pH was selected from the* A*-pH curve.

### 2.9. Determination of Reversible Ability for Taking and Releasing Dioxygen

The reversibility of oxygenation and the kinetics of oxygenation and deoxygenation were carried out with a PP2 flowing injection apparatus [24]. Oxygenation and deoxygenation reversibility of the Co(DABA)_2_ complex was determined by recording absorbance changes at 314 nm upon saturating the solution with O_2_ and N_2_. Absorbance difference, Δ*A*, was used to evaluate O_2_ uptake ability. The number of oxygenation-deoxygenation cycles was used to estimate the endurance to autoxidation of the complex.

### 2.10. Calculation

All calculations were performed with the Gaussian 03W program package [28]. Full geometry optimization computations were performed using the B3LYP method. In all calculations, a LANL2DZ basis set along with the corresponding effective core potential was used for Co metal atoms. The 6-31G basis set was used for C, H, N, and O atoms. The structural models of the studied compounds are shown in Figure 10, and the multiplicity of the complexes was regarded as a quartet for Co(DABA)_2_, a doublet for Co(DABA)_2_O_2_, and a singlet for (DABA)_2_, Co(O_2_), and Co(DABA)_2_.

## 3. Results and Discussion

### 3.1. Formation of the Complex

#### 3.1.1. UV-Vis Spectrophotometry

**Figure 1:**
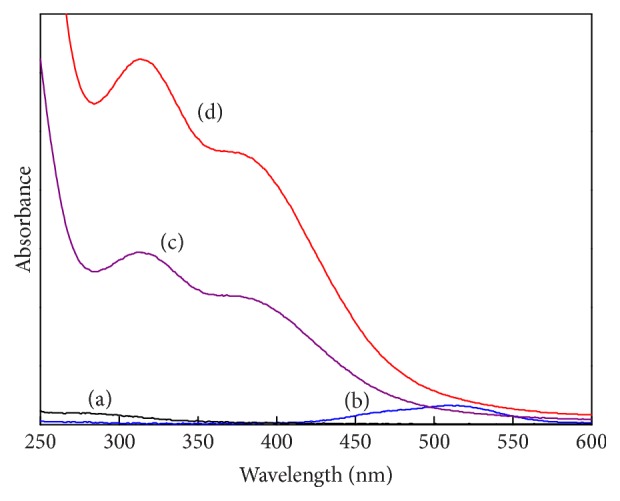
Optical absorption spectra of DABA, Co(II), and complexes in aqueous solution at room temperature (absorption spectra of DABA (a), Co(II) (b), DABA-Co(II) in N_2_ (c), and DABA-Co(II) in O_2_ (d). *C*_DABA_ = 4.0 × 10^−4 ^mol·dm^−3^ and *C*_Co(II)_ = 2.0 × 10^−4 ^mol·dm^−3^).

The optical absorption spectra of the ligand (4.0 × 10^−4 ^mol·dm^−3^) and Co(II) (2.0 × 10^−4 ^mol·dm^−3^) were separately determined. The ligand showed no evident absorption within the range of 200 nm to 600 nm (Figure 1, curve (a)), and Co(II) solutions showed a weak absorption at 520 nm (Figure 1, curve (b)). However, when the ligand and cobalt salt were mixed (v : v = 1 : 1) under N_2_ atmosphere and adjusted to basic conditions, the mixed solution exhibited a yellow color and showed two main absorption peaks at *λ* = 314 and 384 nm. This finding clearly shows the formation of the complex (Figure 1, curve (c)). When N_2_ was replaced with O_2_, the absorption of the solution increased immediately (Figure 1, curve (d)), and the color of the complex solution rapidly changed from light yellow to dark yellow, which indicates that the complex of Co(DABA)_2_ is easily oxygenated.

**Figure 2:**
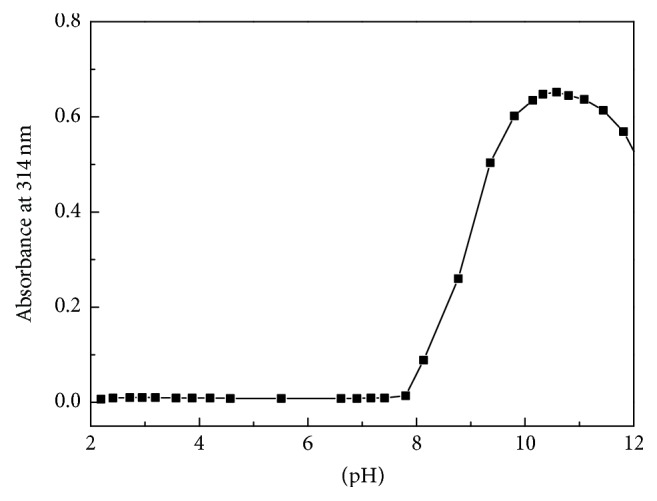
*A*-pH curve of the oxygenated DABA-Co(II) complex (*C*_DABA_ = 4.0 × 10^−4 ^mol·dm^−3^, *C*_Co(II)_ = 2.0 × 10^−4 ^mol·dm^−3^).

According to the* A*-pH curve (Figure 2), complexation started from pH 7.8 and completed at pH 10, as the absorption of the solution reached a maximum in the pH region from 10 to 10.5. Thus, pH 10 was selected for the succeeding tests.

#### 3.1.2. IR Spectroscopy

**Figure 3:**
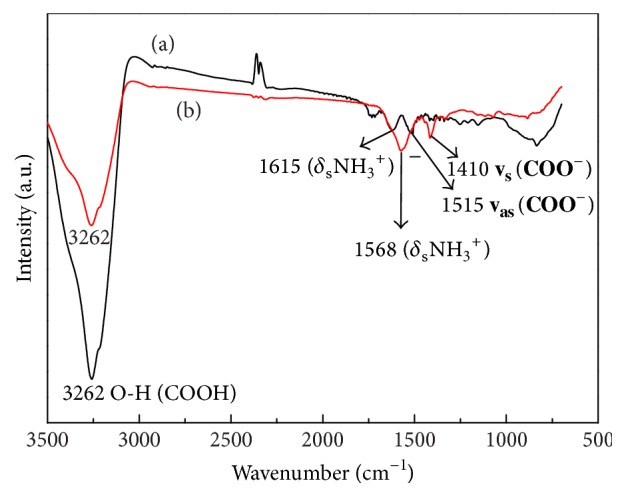
IR spectra of DABA (curve (a)) and DABA-Co(II) (curve (b)) (*C* = 1.0 mol·dm^−3^).

IR characterization of the DABA-Co(II) complex is shown in Figure 3. DABA (Figure 3, curve (a)) presents a strong peak of hydroxyl association at 3262 cm^−1^, as well as two weak peaks of amino stretching vibration and carboxyl asymmetric stretching vibration at 1615 and 1515 cm^−1^, respectively. In the DABA-Co(II) system (Figure 3, curve (b)), when DABA is coordinated with Co(II), the peak at 3262 cm^−1^ becomes weak, the peak at 1515 cm^−1^ disappears, and the peak at 1615 cm^−1^ shifts to red at 1568 cm^−1^. Therefore, the peak changes reveal that the amino group is involved in coordination and show that the ligand DABA and Co(II) form a complex in basic aqueous solution.

#### 3.1.3. Mass Spectrometry

**Figure 4:**
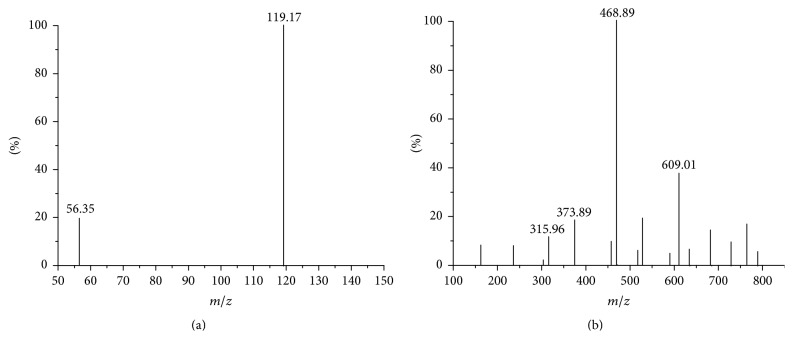
Full-scan positive electrospray mass spectrum of DABA (a) and DABA-Co(II) (b).

The ligand and the DABA-Co(II) complex were analyzed by MS, and the mass spectra are shown in Figure 4. The positive ion mass spectrum of DABA is shown in Figure 4(a). The* m/z* 119.17 fragment was the DABA base peak, which corresponds to the protonated molecule ion [L+H]^+^. In Figure 4(b), the main fragment peaks are* m/z* 609.01, 468.89, 373.89, and 315.96. The peaks could be assigned accordingly to [CoL_2_-O_2_-CoL_2_-2O-2H^+^+Na^+^] or [2(CoL_2_)-2H^+^+Na^+^] (*m/z* 609.01), [Co_2_L_3_-3H] (*m/z* 468.89), [CoL_2_-2H^+^+Ac^−^+Na^+^] (*m/z* 373.89), and [CoL_2_-2H^+^+Na^+^] (*m/z* 315.96). These fragments all confirm the formation of the complex and show that the composition of the complex is CoL_2_.

### 3.2. Composition of the Complex

#### 3.2.1. Molar Ratio Method

**Figure 5:**
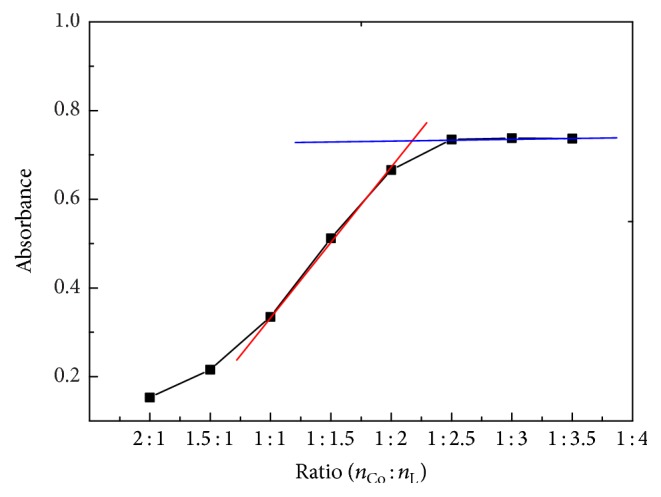
Proportions of the complexes determined by molar ratio method.

The composition of the complex was determined by molar ratio method [29]. The results in Figure 5 show that the intersection of curves occurs at 2 : 1 of the ligand/Co, and the L : Co ratio is confirmed as 2 : 1. These results indicate that the composition of the complex is CoL_2_.

#### 3.2.2. Gas Volumetric Method

**Figure 6:**
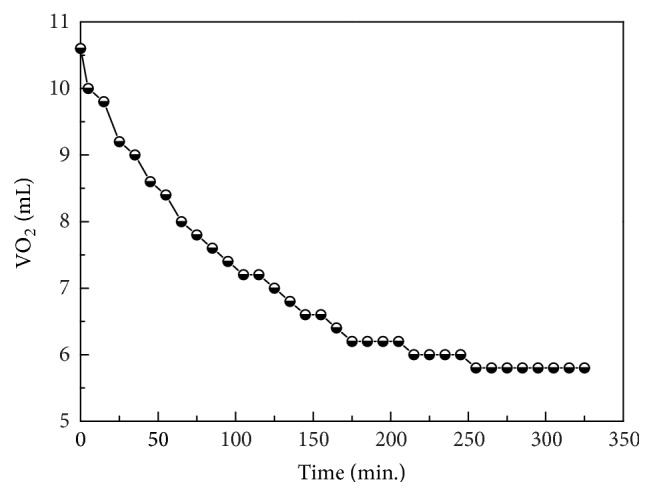
Absorbed volume of oxygen as determined by gas volumetric method.

The capacity of oxygen uptake and the ratio of complex to dioxygen can be determined by volumetry. 10.0 mL of Co(II) (1.0 × 10^−1 ^mol·dm^−3^) and 10.0 mL of DABA (2.0 × 10^−1 ^mol·dm^−3^) were mixed together in a conical flask in a glove box and determined by gas volumetric method [29]. The volumetry results presented in Figure 6 show that 0.5 mmol of Co(DABA)_2_ in aqueous solution can absorb approximately 5.6 mL of oxygen within 5 h. These results reveal that 1 mol of Co(DABA)_2_ can take up 0.5 mol of oxygen and that the composition of the oxygenated complex is (CoL_2_)_2_O_2_. This finding is consistent with the Co/O_2_ ratio in His-Co(II) oxygen complexes [24, 25].

Based on the abovementioned results, we can conclude that the complex forms immediately. In addition, the complex can be easily oxygenated when the two solutions of Co(II) and DABA are mixed and bubbled with dioxygen, and the composition of the oxygenated complex is (CoL_2_)_2_O_2_. Therefore, in the following experiments, the concentration ratio of Co(II) and DABA was considered as 1 : 2, and the ratio of solution volumes was considered as 1 : 1. All tests were conducted rapidly after the two solutions of Co(II) and DABA were mixed and adjusted to pH 10.

### 3.3. Reversible Ability

#### 3.3.1. UV-Vis Spectrophotometry

**Figure 7:**
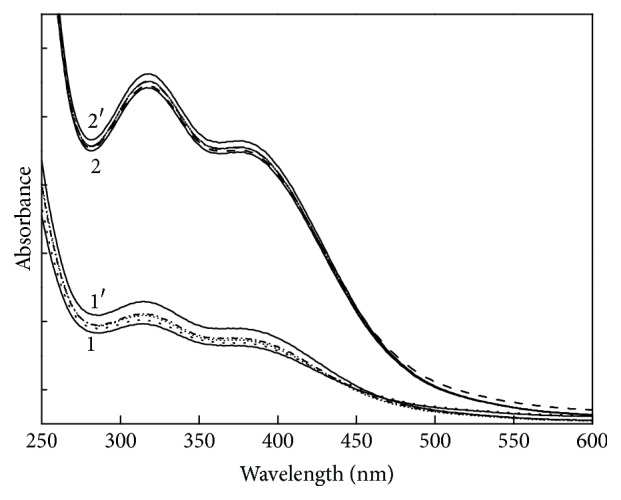
Optical absorption spectra of DABA-Co(II) in aqueous solution and at room temperature. Spectrum 1 was obtained with DABA-Co(II) solution saturated with N_2_. Spectrum 2 was obtained with DABA-Co(II) solution saturated with O_2_. Spectra 1′ and 2′ were obtained on the fifth cycle of alternate bubbling of N_2_ and O_2_ into the DABA-Co(II) solution (*C*_DABA_ = 4.0 × 10^−4 ^mol·dm^−3^ and *C*_Co(II)_ = 2.0 × 10^−4 ^mol·dm^−3^).

The DABA-Co(II) complex is easily oxygenated in aqueous solution at room temperature, and similar oxygenation reaction quickly occurs in air. As shown in Figure 1, the spectra of the original complex and the oxygenated complex are evidently different. Therefore, the UV-Vis spectra can be used to determine oxygenation reversibility. When N_2_ and O_2_ were alternately fed into the DABA-Co(II) solution, the spectra of the solution changed accordingly (Figure 7). After five cycles of N_2_/O_2_ exchanging, both the spectra of the complex in N_2_ and in O_2_ changed slightly, showing that the oxygenation and deoxygenation reactions of the DABA-Co(II) complex are reversible and demonstrating excellent reversibility.

#### 3.3.2. Oxygenmetry

**Figure 8:**
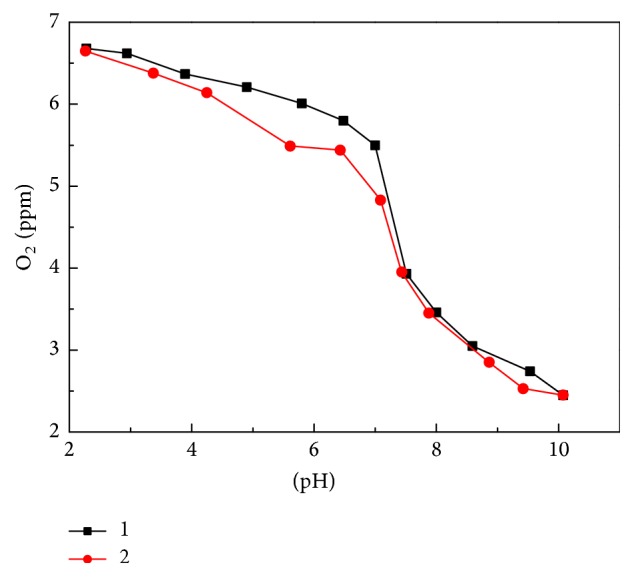
Evolution of dissolved oxygen concentration as a function of solution pH (*C*_DABA_ = 1.2 × 10^−3 ^mol·dm^−3^, *C*_Co(II)_ = 6.0 × 10^−4 ^mol·dm^−3^).

Oxygenmetry was used to detect dioxygen concentration in the solution to follow the oxygenation evolution of the complex. As shown in curve 1 of Figure 8, dioxygen concentration dropped sharply after pH 7 and reached a plateau near pH 10 with the increase in pH. This finding suggests that the complex started to be oxygenated at pH 7 and that the reaction completed at pH 10. The pH of this solution was then adjusted from 10 to 2 with dilute HCl. It can be seen that curve 2 almost coincided with curve 1, indicating that the oxygenation of the complex is reversible.

#### 3.3.3. Reversibility of Dioxygen Uptake and Release

**Figure 9:**
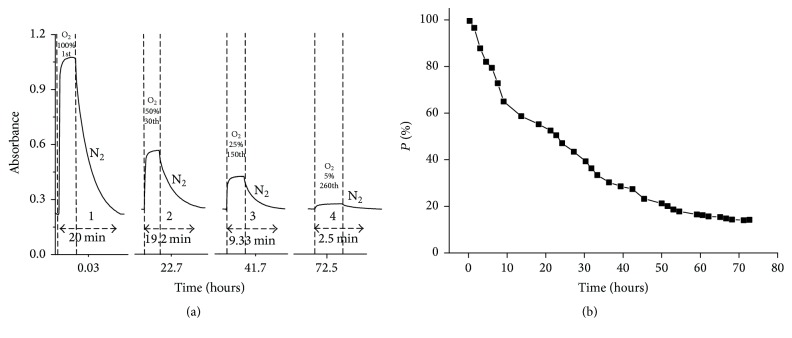
(a) Absorbance changed at 314 nm as N_2_ and O_2_ were bubbled alternately through an DABA-Co(II) complex aqueous solution (*C*_Co(II)_ = 2.0 × 10^−4 ^mol·dm^−3^). (b) Percentage of oxygenation capacity (*P*%) of DABA-Co(II) as a function of oxygenation time.

To investigate the durability of the DABA-Co(II) complex according to O_2_ uptake, we conducted oxygenation-deoxygenation of DABA-Co(II) continuously by purging O_2_ and N_2_ alternately in aqueous solution at 25°C ± 0.1°C. Our results show that, by alternately changing N_2_ and O_2_, oxygenation-deoxygenation cycles could be conducted continuously for 3 days and sustained for 260 cycles (Figure 9). It can be observed that oxygen uptake of the complex occurs rapidly and is completed in 2 min (Figure 9(a) (curve 1)). However, the deoxygenation rate is relatively slow; it took 15 min to fully release the absorbed dioxygen.

**Scheme 1:**
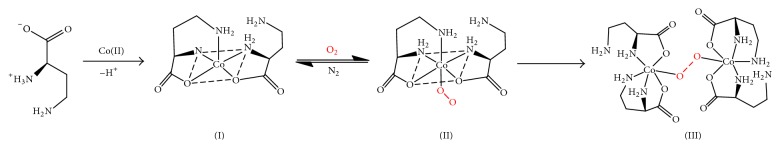
Oxygenation mechanism for DABA-Co(II) complex with dioxygen.

The rate constant for oxygenation (*k*_*a*_) of Co(DABA)_2_ was calculated using optical absorption data averaged from numerous individual cycles (Figure 9(a)). Data analysis suggest that the oxygenation reaction is a first-order reaction, with *k*_*a*_ = 0.0139 s^−1^, whereas deoxygenation reaction is more complicated. For this complex, the experiment demonstrates that the mechanism for the oxygenation reaction is SN1 while the mechanism for deoxygenation reaction is SN2. In the oxygenation reaction, the Co ion in Co(DABA)_2_ turned from a high spin state into low spin states. Therefore, the ion can combine with oxygen at the axial coordinate space through a one-step reaction to generate oxygenated complexes (Scheme 1). Meanwhile, the ion is converted into low spin states, in which the ligand-binding ability increases. Thus, the removal of oxygen becomes much more difficult, and a solvent (water) molecule was involved as a competitive ligand to crowd oxygen out to form a nonoxygenated complex, which results in a SN2 reaction. This result is similar to that of*β*-(2-Pyridyl)-*α*-alanine [24].

#### 3.3.4. Oxygenation Capacity

The reversibility of oxygenation and deoxygenation of Co (DABA)_2_ was determined by monitoring the change in absorbance at 314 nm upon saturating the solution with O_2_ and N_2_, respectively. The absorbance difference Δ*A* was used to evaluate O_2_ uptake capacity, and the number of oxygenation-deoxygenation cycles was used to estimate the endurance of the complex against autooxidation. Take Δ*A* = *A*_O_2__ − *A*_N_2__ and first Δ*A* as Δ*A*_0_: *P*% = Δ*A*/Δ*A*_0_ *∗*100%. The percentage of oxygenation capacity (*P*%) was used as a function of oxygenation-deoxygenation cycle to evaluate the endurance of the complexes against autooxidation.

Figure 9 presents the decline trend of the oxygenation capacity of DABA-Co(II). The figure shows that the DABA-Co(II) complex maintained 50% of its original oxygenation capacity after 30 cycles nearly in one day. Afterward, degradation slowed down, and 5% of the original oxygenation capacity remained after more than 260 cycles and 3 days. This result proves the excellent oxygenation-deoxygenation durability of DABA-Co(II) complex [14, 15].

#### 3.3.5. DFT Calculation

**Figure 10:**
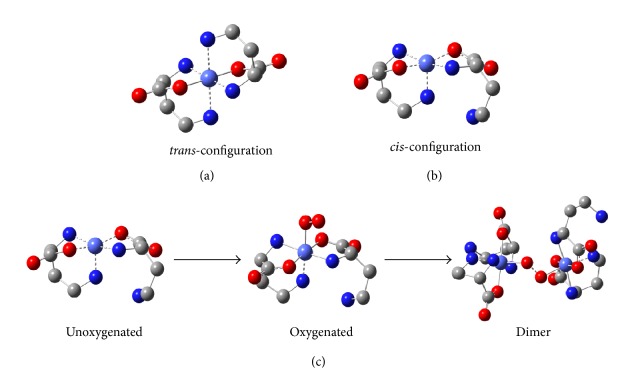
((a), (b)) Two proposed deoxygenation configurations of Co(DABA)_2_ and (c) simulation of oxygenation of Co(DABA)_2_.

To clarify the oxygenation and deoxygenation of the DABA complexes, we carried out DFT calculations using the G03 quantum chemistry software [29]. The structural models of the studied compounds are shown in Figure 10. For the deoxygenated configuration of Co(DABA)_2_, we proposed two kinds of configuration, as shown in Figure 10. The first configuration is in a* trans*-configuration with a 4-amino in* para*-position (Figure 10(a)), and the second configuration is in a* cis*-configuration (Figure 10(b)). The calculation results show that the energy levels of* cis*-configuration are lower than those of the* trans*-configuration (Δ*G* = −228.95·kJ mol^−1^). This finding indicates that the* cis*-configuration is the main species in the solution. The results are similar to those of bis[b-(2-pyridyl)-a-alaninato]-Co(II), [PyA-Co(II)] [24, 30].

During the formation of the oxygenated complexes, the electron of *e*_*g*_ orbitals of Co(II) would transfer to *π* ^*∗*^ orbitals of dioxygen to form the Co-O_2_ bond. For the* cis*-configuration, the structure is almost unaltered, and only dioxygen occupies the originally unoccupied position. The dioxygen bond length increased from 0.1207 nm of the free state to 0.1359 nm in the coordinated state. These values are similar to the calculation values (from 0.1207 nm to 0.1357 nm) of PyA-Co(II) [24], indicating that oxygenation and deoxygenation are similar to those of the PyA-Co(II) system. However, by comparing the dimer oxygenation species in both systems, we noticed that the bond length of O-O was different. The dioxygen bond length is 0.1493 nm for DABA-Co(II) system and 0.1454 nm for PyA-Co(II) system. Hence, the complex is prone to forming dimer oxygenation species and easily producing an irreversible oxidation, and oxygenation reversibility decreased. Therefore, the oxygenation reversibility of Co(DABA)_2_ is slightly lower than that of the PyA-Co(II) system, despite the similar coordination structures of the two complexes.

Based on the simulated oxygenation configuration, we here propose model which supports 4-amino dissociates in one ligand for* cis*-configurations. This 4-dissociated amino group may form bridging coordination with another Co(DABA)_2_ complex, leading to the emergence of [Co_2_(DABA)_3_] and [Co(DABA)_2_]_2_ species in MS determination.

## 4. Conclusion 

The oxygenation behavior of the DABA-Co(II) complex in aqueous solution was investigated. The formation of the complex was confirmed by UV-Vis, IR, and MS characterization, and the composition of the complex was determined by gas volumetric method, molar ratio method, and MS. The results indicate that the proportion of oxygenated complex is* n*(Co) :* n*(DABA) :* n*(O_2_) = 1 : 2 : 0.5. The reversibility of oxygenation and deoxygenation of the complex in aqueous solution was tested with oxygenmetry and UV-Vis, and both results confirm that the oxygenation process is a reversible reaction. Furthermore, the complex exhibit excellent oxygen uptake ability and can continuously absorb and release oxygen for 3 days by alternately changing N_2_ and O_2_, over 260 cycles. The excellent reversible oxygenation property of the complex further confirmed our assumption that a Co(II) complex with a histidine analogue ligand uptakes oxygen reversibly. The study on relationship between structural detail and oxygenation properties will help to explore new families of oxygen carriers.
